# The role of empiric atypical antibiotic coverage in non-severe community-acquired pneumonia

**DOI:** 10.1017/ash.2024.453

**Published:** 2024-12-11

**Authors:** Whitney Hartlage, Hannah Imlay, Emily S. Spivak

**Affiliations:** 1 Division of Infectious Diseases, Veteran’s Affairs Salt Lake City Health Care System, Salt Lake City, UT, USA; 2 Division of Infectious Diseases, Department of Internal Medicine, University of Utah, Salt Lake City, UT, USA

## Abstract

A β–lactam plus a macrolide or a respiratory fluoroquinolone alone is recommended as standard empiric antibacterial therapy for non-severe adults hospitalized with community-acquired pneumonia (CAP) per Infectious Diseases Society of America guidelines. However, the evidence in support of adding empiric atypical antibacterial therapy, and specifically the addition of a macrolide, is conflicting and should be balanced with additional factors: the necessity of covering atypical organisms, benefits of macrolide-associated immunomodulation, harms associated with antibiotic use, and selection for antibiotic-resistant organisms. In this review, we examine the role of atypical coverage in standard treatment regimens for patients admitted with non-severe CAP and specifically focus on the addition of macrolides to β–lactams. We conclude that a subset of patients should not be given atypical coverage as part of their regimen.

## Introduction

Community-acquired pneumonia (CAP) is a leading cause of hospitalization in the United States and a common indication for antimicrobials in the inpatient setting.^1,2^ Guidelines suggest standard empiric treatment strategies should target the most common bacterial causes of CAP and vary depending on severity of illness. Currently, a β–lactam plus a macrolide or a respiratory fluoroquinolone alone is recommended as initial treatment for all hospitalized patients admitted with non-severe CAP in North America.^3^ Meanwhile, British guidelines reserve agents with atypical bacterial coverage, defined as antibacterials that cover the atypical pathogens *Mycoplasma pneumoniae, Chlamydia pneumoniae*, and *Legionella* species and typically include macrolides, respiratory fluoroquinolones, or tetracyclines, to those with moderate to high pneumonia severity.^4^ Due to conflicting data on clinical outcomes and the changing epidemiology of CAP,^5^ the need to routinely cover atypical pathogens remains a subject of debate.

This review focuses on the origin, rationale, and data guiding the addition of empiric atypical antibacterial therapy for patients hospitalized with non-severe CAP. For the purposes of this review, we will focus on the addition of a macrolide to β–lactam therapy and define non-severe CAP as non-intensive care unit patients admitted to a general medical ward. In this review, we consider *Legionella* species the primary target of atypical antibacterial coverage among hospitalized adults. We additionally highlight factors that should be considered when designing guidelines for CAP and the selection of empiric atypical antibacterial therapy.

## Origin of current recommendation

The first guidelines endorsed by the Infectious Diseases Society of America (IDSA) for CAP were published in April 1998.^6^ Due to the limitations of diagnostic testing and the inability to adequately identify pneumonia pathogens, initial treatment recommendations were largely empiric and directed towards the most common pathogens outlined at the time: *Streptococcus pneumoniae, Haemophilus influenzae,* and atypical organisms (*M. pneumoniae, C. pneumoniae*, and *Legionella* species).^7^ The suggested regimen for hospitalized patients admitted to the general medicine ward included a β–lactam, with or without a macrolide, or a fluoroquinolone alone. Due to the absence of well-designed prospective studies, the authors acknowledged that this initial recommendation was largely based on clinical experience and/or in vitro activity, contributing to its B-II recommendation.

Subsequent versions of the IDSA guidelines for CAP in adults were updated in 2000, 2003, and 2007.^8–10^ Variations in guideline iterations and the evidence to support the recommendation for combination β–lactam-macrolide therapy are summarized in Table 1. Notably, after the initial 1998 Guideline was published, retrospective data and Medicare database evaluations emerged that suggested the addition of a macrolide to a β-lactam lowered mortality compared to a β-lactam alone. As a result of these observational study findings, the option of β–lactam monotherapy changed to recommend a β-lactam PLUS a macrolide or a fluoroquinolone alone.^8^ Additionally, in the 2003 Guidelines, the level of evidence for the β–lactam-macrolide combination recommendation was upgraded from a B-II to an A-I; however, no randomized controlled trials (RCT) were referenced to support the recommendation increase or to suggest combination therapy with a macrolide was superior to monotherapy with a β-lactam.^9^

**Table 1.** Differences in IDSA Guideline recommendations for non-severe/non-ICU inpatients hospitalized with CAP and the supporting evidence

Guideline/Recommendation	Level of Evidence	Evidence to Support Recommendation
**1998 IDSA Guideline**^6^ β-lactam **with or without** a macrolide or a fluoroquinolone alone	B-II	Per the authors, “the selection of these regimens are based largely on clinical experience and/or in vitro activity.”
**2000 IDSA Guideline**^8^ β-lactam **PLUS** a macrolide or a fluoroquinolone alone	B-II	Based on a retrospective evaluation of nearly 13,000 Medicare database patients that found the addition of a macrolide to a β-lactam was associated with a lower 30-day mortality compared to a β-lactam alone.^56^ Due to the limitations, the authors acknowledged that future randomized controlled trials would be needed to confirm these findings before they are adopted into clinical practice.
**2003 IDSA Guideline**^9^ β-lactam **PLUS** a macrolide or a fluoroquinolone alone	A-I	Recommendation remained the same, however, no randomized controlled trials noted in guidelines to support level of evidence switch from B-II to A-I.
**2007 ATS/IDSA Guideline**^10^ β-lactam **PLUS** a macrolide or a fluoroquinolone alone	Strong recommendation, level I evidence	The authors state “the recommendation of combination treatment is based on retrospective studies demonstrating a significant reduction in mortality compared with that associated with a cephalosporin alone.” The additional studies cited to support this recommendation compared to prior versions include two retrospective studies of patients using Medicare/hospital claims-made databases and an observational study that aimed to characterize the “real world” treatment of CAP by surveying nonteaching US community hospitals.^56–59^
**2019 ATS/IDSA Guideline**^3^ β-lactam **PLUS** a macrolide or a fluoroquinolone alone	Strong recommendation, high quality of evidence	The recommendation for combination therapy is based on: 1) a RCT for which the authors “could not rule out the possibility that β–lactam monotherapy was inferior to β–lactam-macrolide combination therapy”, 2) a systematic review of 20 experimental and observational studies in adults hospitalized with CAP that found β–lactam-macrolide combination or fluoroquinolone monotherapy was generally associated with lower mortality than β–lactam monotherapy, 3) a systematic review consisting of several cohort and retrospective observational studies that found combination therapy reduced mortality in patients with CAP compared to monotherapy, and 4) a systematic review consisting of two RCTS and many observational studies that demonstrated β–lactam-macrolide combination therapy in patients with CAP may decrease all-cause death compared to β–lactam monotherapy, but only for patients with severe CAP.

ATS, American Thoracic Society; IDSA, Infectious Diseases Society of America; CAP, community-acquired pneumonia; RCT, randomized controlled trial; US, United States.

In summary, the origin of empiric combination therapy with a macrolide in hospitalized patients was derived from observational studies, in vitro data, and expert opinion. Since these initial guideline iterations, well-designed RCTs have been conducted to address the role of empiric atypical antibacterial coverage in CAP. Despite this, the current 2019 IDSA guideline recommendation for combination therapy in non-severe CAP seems to be supported by observational studies and a systematic review of experimental and observational data. Guideline authors cite one RCT for which they acknowledge they “could not rule out the possibility that β-lactam monotherapy was inferior to β-lactam-macrolide therapy for inpatients with CAP^3^.” We thus urge clinicians to better understand the level of evidence that established this historical practice and call for a reevaluation of this topic in future guidelines and clinical practice in light of newer, high-quality outcome data. Data evaluating the role of empiric atypical antibacterial coverage should also be balanced against additional factors, such as the changing epidemiology of CAP, advances in diagnostic methods, harms associated with antibacterials, and increased antibiotic resistance.

## Diagnostic accuracy and evolving etiology of pneumonia

Published data has increased our understanding of the poor diagnostic accuracy and evolving microbiologic causes of pneumonia. These findings should further inform the role of empiric atypical antibacterial coverage in hospitalized adults with non-severe CAP.

The diagnosis of pneumonia is challenging. Presenting signs and symptoms can vary among patients and there are many conditions that can mimic pneumonia, including pulmonary edema, malignancy, interstitial lung disease, eosinophilic pneumonia, diffuse alveolar hemorrhage, and pulmonary embolism, among others.^11^ Several studies demonstrate the diagnostic uncertainty of hospitalized patients with a diagnosis of CAP.^12–14^ First, a single-center prospective study conducted by Musher and colleagues found that 17% (n = 44/259) of patients hospitalized with a CAP diagnosis and treated with antibiotics were determined to not be infected.^12^ Similarly, Gupta and colleagues performed a multicenter prospective study across 48 Michigan hospitals and found that 12% (n = 2,079/17,290) of hospitalized adults treated for CAP were inappropriately diagnosed.^14^ As a result, these studies illustrate the inappropriate diagnosis of CAP is common and as many as 1 in every 6 to 8 adults admitted for pneumonia may not benefit from antibacterial coverage, regardless of agent selection.

Historical surveillance data conducted in the 1990s and prior to the availability of more sensitive molecular and antigen-based testing suggested *M. pneumoniae, C. pneumoniae*, and *Legionella* species together accounted for 10%–38% of pneumonia cases in hospitalized adults.^5,7^ However, recent availability and increased uptake of improved diagnostic testing methods for respiratory infections has greatly enhanced our ability to quickly detect viral and bacterial pathogens, including atypical organisms.^15^ With these advancements, the detection of atypical pathogens in patients with a clinical diagnosis of pneumonia is far less than what was reported in the past (Table 2).^5,12,16–18^ In the Center for Disease Control and Prevention’s EPIC study, adults with radiographic evidence of pneumonia across five hospitals in the United States underwent robust bacterial and viral testing to investigate the microbiologic causes of CAP requiring hospitalization.^5^ Of the 2,320 adults, nasopharyngeal and oropharyngeal swabs were obtained from 98% (for the detection of multiple viruses plus *C. pneumoniae* and *M. pneumoniae),* blood culture from 91%, a specimen for urinary antigen detection (pneumococcal and *Legionella*) from 85%, and a sputum specimen from 41%. *Legionella pneumophila* was detected in 1% of cases and *Mycoplasma/Chlamydia* together accounted for <3% of cases. Respiratory viruses were detected more frequently than bacteria (27% versus 14%), and despite the thorough microbiology testing, no pathogen was detected in the majority of patients (62%).

**Table 2.** Etiology of community-acquired pneumonia (CAP), studies since 2010^a,*^

	Study Location			
Country	US^12^	US^5^	Netherlands^16,17^	Greece^18^
Number of patients with CAP	215	2,320	1,240	267
Pathogens^b^				
Bacteria	29%	15%	22%	69%
*S. Pneumoniae*	9%	5%	14%	6%
*Haemophilus influenzae*	6%	<1%	3%	15%
*S. Aureus*	4%	2%	1%	20%
*Pseudomonas aeruginosa*	3%	<1%	1%	6%
*Legionella*	1%	1%	1%	1%
*Mycoplasma, Chlamydia*	-	<3%	1%	-
Other bacteria	6%	3%	5%	-^d^
Mycobacteria	2%	1%	<1%	-
Nocardia	1%	0%	0%	-
Fungi^c^	3%	1%	3%	-
Viruses	20%	27%	14%	10%
Rhinovirus	12%	9%	3%	4%
Coronavirus	3%	2%	2%	-
Human metapneumovirus	2%	4%	1%	<1%
Influenza	1%	6%	3%	2%
Parainfluenza	2%	3%	-	1%
RSV	1%	3%	1%	2%
Other viruses	-	2%	-	-^d^
No cause identified	55%	62%	74%	46%

a This table was adapted from the 2019 CAP Guideline Supplement.

b Patients with confirmed bacterial and viral co-infection are listed in each column.

c *Pneumocystis jiroveci*.

d Authors did not differentiate other bacteria versus other viruses.

Microbiological findings from these studies highlight that a proportion of patients requiring hospitalization for CAP would not respond to standard empiric treatment recommendations. For example, *Pseudomonas, Mycobacteria, Nocardia*, and fungi were all detected at rates similar to or higher than atypical pathogens.^5,12,18^ However, in the absence of risk factors, initial treatment strategies targeting these pathogens are not routinely recommended for all inpatient adults with non-severe CAP.^2^ Given the rare incidence of *M. pneumoniae, C. pneumoniae*, and *Legionella* species combined with diagnostic uncertainty, it seems a similar clinical approach is reasonable given the unclear benefit of empiric atypical antibacterial coverage for all patients with non-severe CAP presentations.

## Clinical trial data evaluating the addition of empiric atypical antibacterial coverage in non-severe hospitalized patients with CAP

Several meta-analyses have examined the impact of empiric atypical coverage, although the majority of included studies did not directly compare β-lactam monotherapy to β-lactam-macrolide combination therapy (atypical coverage was often provided by a fluoroquinolone). In 2012, a Cochrane review of 28 RCTs by Eliakim-Raz et al estimated the risk of mortality and treatment failure among hospitalized patients with CAP^19^ and found no difference in mortality (RR 1.14; 95% CI 0.84–1.55) or clinical success (RR 0.93; 95% CI 0.84–1.01) associated with regimens that included atypical coverage; this finding was consistent with other earlier meta-analyses as well.^20,21^ To address limitations of other meta-analyses, Eljaaly and colleagues conducted a newer meta-analysis of five RCTs to evaluate the impact of atypical coverage on rates of clinical failure using more stringent inclusion criteria.^22^ Although the included studies were unable to identify a difference in the efficacy outcome individually, a lower clinical failure rate was observed with empiric atypical coverage (RR 0.85; 95% CI 0.73–0.99) when studies were combined. However, the absolute difference in clinical failure was small (22% in the atypical arm versus 26% in the non-atypical arm), and there was no difference in mortality (RR 0.55; 95% CI 0.26–1.17). It is worth noting significant heterogeneity among the five included RCTs exists limiting the ability to evaluate which individuals may derive maximal benefit with empiric atypical coverage. For example, one study excluded patients with pneumonia suspected due to an atypical pathogen, multiple studies included patients with severe CAP (2 of 5), and 4/5 of the included RCTs were not evaluating the role of empiric atypical coverage versus non-atypical coverage and instead were comparing the efficacy of fluoroquinolones to β-lactams for CAP.

Three RCTs evaluating the role of empiric β-lactam-macrolide combination therapy versus β-lactam monotherapy for hospitalized patients with CAP have been conducted (Table 3).

**Table 3.** Studies comparing antibiotic regimens with atypical coverage to regimens without atypical antibiotic coverage among adults hospitalized with community-acquired pneumonia (CAP)

Study	Study design and population	Intervention	Primary outcome	Results
Eliakim-Raz, et al.^19^	Meta-analysis including 28 RCTs and adult patients hospitalized due to CAP (n = 5,939)	Atypical coverage vs. regimen without atypical coverage	Mortality and proportion of treatment failure between regimens	No difference in mortality (RR 1.14; 95% CI 0.84–1.55) or clinical success (RR 0.93; 95% CI 0.84–1.01) between the atypical arm and the non-atypical arm
Garin, et al.^20^	Open-label, noninferiority RCT in adult patients with moderately severe CAP (n = 580)	β-lactam monotherapy vs. β-lactam + macrolide	Proportion not meeting clinical stability by day 7	-Noninferiority of β-lactam monotherapy to β-lactam + macrolide was not shown (41.2% in the monotherapy arm versus 33.6% in the combination arm; absolute difference 7.6%, p=0.7) -Superiority of the β-lactam-macrolide combination was also not shown -Time to clinical stability did not differ by intervention arm for patients without atypical pathogens or in those in lower PSI categories (I-III) -No difference in intensive care unit admissions, length of hospital stays, mortality, 90-day readmission, or new pneumonia within 30 days
Postma, et al.^21^	Cluster-randomized, crossover, noninferiority trial in adults admitted to non-intensive care until hospital wards (n = 2,283)	β-lactam monotherapy vs. β-lactam + macrolide vs. Fluoroquinolone monotherapy	All-cause mortality at 90 days	-No difference in 90-day mortality -β–lactam monotherapy was noninferior to treatment with β–lactam-macrolide combination therapy (Absolute difference, 1.9%; 90% CI, –0.6–4.4) and fluoroquinolone monotherapy (Absolute difference –0.6%; 90% CI, –2.8–1.9) -No differences in length of hospital stay or complications were reported among treatment strategies
Giamarellos-Bourboulis, et al.^18^	Prospective, double-blind, RCT in non-ICU adults hospitalized with CAP and features of sepsis (n = 267)	β-lactam monotherapy vs. β-lactam + macrolide	Composite endpoint: ≥50% decrease in respiratory symptom severity score from baseline and a ≥30% decrease in SOFA score from baseline or favorable change in procalcitonin kinetics (≥80% decrease in procalcitonin from baseline or value <0.25 ng/mL) at day 4	-Composite primary endpoint was met in 68% of the β-lactam-macrolide arm versus 38% in the β-lactam monotherapy arm (difference 29.6%; 95% CI, 17.7–40.3) -No difference in mortality by days 28 and 90

RCT, randomized controlled trial; CAP, community-acquired pneumonia; PSI, pneumonia severity index; ICU, intensive care unit; SOFA, sequential organ failure assessment.

In 2014, Garin and colleagues conducted a noninferiority randomized trial that compared antibiotic treatment with a β–lactam alone (n = 291) to a β–lactam-macrolide combination (n = 289) in non-ICU patients with moderately severe CAP (Pneumonia Severity Index (PSI) category I-IV).^23^ The primary outcome was the proportion of patients who did not reach clinical stability at day 7, defined as having to achieve and maintain all five of the following criteria for a minimum of 24 hours: heart rate <100/min, systolic blood pressure >90mmHg, temperature <38.0 C, respiratory rate <24/min, and oxygen saturation by pulse oximetry of more than 90% on room air. Noninferiority of β–lactam monotherapy to β–lactam-macrolide combination therapy was not shown (patients not reaching clinical stability at day 7 was 41.2% in the monotherapy arm versus 33.6% in the combination arm; absolute difference 7.6%, *P* = 0.07). Patients infected with atypical pathogens or more severe disease (PSI category IV pneumonia) appeared to benefit the most from combination therapy with a macrolide. Time to clinical stability did not differ between study arms for patients without atypical pathogens or those in lower PSI categories (I–III). There was no difference in intensive care unit admissions, length of hospital stays, mortality, 90-day readmission, or new pneumonia within 30 days between the two arms. Notably, the primary outcome was evaluated at day 7; however, the median time to achieve clinical stability by study definition was 9.5 days in monotherapy arm vs. 8.5 days in combination therapy arm (*P* = 0.44) and longer than previous reports. The authors acknowledged that despite randomization, there was an imbalance in the distribution of patients infected with *Legionella* between treatment arms, which could have favored the combination arm (*Legionella* species were detected in 4.1% of the monotherapy arm versus 1.4% of combination therapy arm). Lastly, to further explore the potential non-antibacterial effect of macrolides, a subgroup analysis excluding patients with atypical pathogens found the trend towards a better outcome in the combination arm persisted (absolute difference in the primary outcome was 5.8% compared to 7.6% in primary analysis), suggesting any benefit of the macrolide was possibly mediated through a non-antibacterial mechanism.

Subsequently, Postma and colleagues conducted a cluster-randomized, crossover, noninferiority trial in 2015 comparing β–lactam monotherapy (n = 656) to treatment strategies consisting of β–lactam-macrolide combination therapy (n = 739) or fluoroquinolone monotherapy (n = 888) in patients with clinically suspected CAP who were admitted to non-ICU hospital wards.^24^ When evaluating all-cause mortality at 90-days, empiric treatment with β–lactam monotherapy was noninferior to treatment with β–lactam-macrolide combination therapy (absolute difference, 1.9%; 90% CI, –0.6–4.4) and fluoroquinolone monotherapy (absolute difference, –0.6%; 90% CI, –2.8–1.9). No differences in length of hospital stay or complications were reported among treatment strategies.

Lastly, in 2024, Giamarellos-Bourboulis and colleagues published results of the randomized, double-blind, placebo-controlled trial, known as ACCESS, which explored the anti-inflammatory role of macrolides in CAP management.^18^ The ACCESS trial randomized non-ICU patients hospitalized with CAP and features of sepsis to β–lactam monotherapy (n = 133) or β–lactam-clarithromycin combination therapy (n = 134). Enrolled patients were required to meet all the following: ≥2 systemic inflammatory response syndrome criteria, a Sequential Organ Failure Assessment (SOFA) score ≥2, and a procalcitonin level ≥0.25 ng/mL. The composite primary endpoint at day 4 required patients to have a ≥50% decrease in respiratory symptom severity score from baseline and a ≥30% decrease in SOFA score from baseline or favorable change in procalcitonin kinetics (≥80% decrease in procalcitonin from baseline or value <0.25 ng/mL), and was met in 68% of the β–lactam-clarithromycin arm versus 38% in the β–lactam monotherapy arm (absolute difference 29.6%, *P* <0.001). Mortality at days 28 and 90 did not differ between study arms. Notably, isolation of atypical pathogens was low; one patient (1%) in the clarithromycin group and three (2%) patients in the placebo group were infected with *Legionella*. Although the authors found low rates of atypical infection and no impact on mortality, results of the ACCESS trial suggest empiric combination therapy with a macrolide in non-ICU CAP patients who present with more severe disease may expedite resolution of symptoms. The impact of time to clinical resolution on downstream clinical outcomes is unclear and requires further study.

In summary, we could not identify any high-quality or randomized studies suggesting a survival benefit with the addition of empiric atypical antibacterial coverage in non-severe hospitalized patients with CAP. The data that exists suggests patients with PSI category IV pneumonia may have a quicker time to clinical stability with the addition of a macrolide to β–lactam therapy; however, the lack of improvement in clinical outcomes identified among all non-ICU hospitalized CAP patients suggests it is reasonable to withhold empiric atypical antibacterial coverage in the majority of those with non-severe CAP in the absence of more severe presentations. These studies also confirm that atypical pathogens are detected in a small proportion of patients with confirmed CAP.

## Diagnostic considerations

Due to the limitations in performance characteristics, inadequate availability, increased costs, and delayed turnaround time of classical diagnostic methods, clinicians were previously unable to confirm or exclude the diagnosis of specific respiratory pathogens in a timely fashion, leaving treatment of CAP to be largely empiric.^6^ However, the increased availability and uptake of various testing methods within the last decade has increased access to sensitive diagnostic tests with rapid turnaround time for common bacterial and viral causes of CAP, including *M. pneumoniae, C. pneumoniae*, and *L. pneumophila*.^15^ Therefore, in patients presenting with non-severe CAP, withholding macrolides from standard empiric treatment regimens and tailoring therapy if and/or when these infections are identified may be a more stewardship-minded approach for this syndrome.

Two of the RCTs referenced above support this approach.^23,24^ For example, in the study conducted by Garin and colleagues,^23^ a macrolide was subsequently added to the patients in the β–lactam monotherapy arm if they were found to be infected with an atypical pathogen. In these patients, there was no statistical difference in in-hospital death, intensive care unit admissions, complicated pleural effusion, length of stay, and 30-day or 90-day death or readmission compared to patients with confirmed atypical infection who received initial β–lactam-macrolide combination therapy. Similarly, in the study conducted by Postma and colleagues,^24^ 1% (n = 5/492) of the patients who underwent urinary testing for *Legionella* tested positive; two received ciprofloxacin empirically due to the high clinical suspicion and three had therapy adjusted after the test result. The authors noted that all five had a good clinical outcome. These studies suggest empiric atypical antibacterial therapy may not be necessary given treatment initiated based on diagnostic results was not associated with worse outcomes.

Lastly, the availability of *Legionella*-specific testing impacts the confidence of a non-*Legionella* diagnosis. Even in the absence of *Legionella* testing, we believe that empiric addition of a macrolide to β–lactam therapy is still unnecessary in patients with non-severe CAP given the low prevalence of these infections. However, in resource-limited settings where the availability of antigen-based or molecular testing is limited, we acknowledge that treatment in these situations remains largely empiric and upfront combination therapy including a macrolide may be reasonable.

## Increased understanding of the harms associated with macrolide use

A judicious review of the evidence supporting guideline recommendations should be paired with antimicrobial stewardship principles when incorporating guidance into clinical practice. As new evidence on antimicrobial resistance and harms associated with antimicrobial use becomes available, the applicability of guideline recommendations should be reevaluated to balance benefits with potential risks.

Excessive macrolide use has led to an increase in antimicrobial resistance among various pathogens. First, macrolide resistance has been identified in nearly 40% of *S. pneumoniae* isolates throughout inpatient and ambulatory care settings.^25–27^ Second, studies suggest macrolide resistance has become a concern for many other organisms that were originally susceptible, including *M. pneumoniae*, *Staphylococcus* spp., non-*S. pneumoniae Streptococcu*s spp., *N. gonorrhoeae*, *M. genitalium*, *T. pallidum*, *P. acnes*, *Campylobacter* spp., and *Enterococcus* spp., among others.^28–40^

In addition to the concerning resistance trends, azithromycin use is associated with adverse events. In 2012, a study by Ray and colleagues found a 2.9-fold higher risk of cardiovascular death within 5 days of azithromycin therapy compared to amoxicillin therapy, which was most pronounced among patients with baseline risk of cardiovascular disease.^41^ In a subsequent large cohort study consisting of young and middle-aged adults with a relatively lower baseline risk of cardiovascular disease (mean age of cohort was 40 years), the authors failed to detect an increased risk of death from cardiovascular causes.^42^ As a result of its identified proarrhythmic effects among those with cardiovascular disease, the US Food and Drug Administration (FDA) issued a warning in 2013 cautioning the use of azithromycin in those with known cardiac risk factors, including existing QT interval prolongation, torsade de pointes, electrolyte imbalances, bradycardia, or concomitant use of medications that prolong the QT interval.^43^ Despite this FDA warning and that older adults both have a high rate of pre-existing chronic cardiac conditions and represent the majority of admissions due to pneumonia, macrolides remain recommended in standard treatment regimens for all inpatients.^44–46^ Additionally, although these agents are typically prescribed for short treatment durations, an additional review evaluating antibiotic harms also found that macrolides were associated with significant increases in the odds of developing an adverse event with each day of therapy (OR 1.05, 95% CI 1.01–1.10).^47^

Collectively, the risks associated with routine use of macrolides for common diagnoses should be weighed against the potentially low utility of these agents in most adults admitted with a CAP diagnosis. The desirability of outcome ranking trial design was developed to assess the risks and benefits between various treatment strategies.^48^ This novel approach should be considered for future studies to address the role of empiric atypical coverage in non-severe hospitalized patients with CAP to more comprehensively inform overall impact integrating benefits and harms.

### Which patients may benefit from upfront combination therapy with a macrolide for CAP?

Data supports combination therapy with a macrolide as empiric management for several groups of patients with CAP. First, patients presenting with CAP with a high clinical suspicion for *Legionella*, including those presenting from an area of a known *Legionella* outbreak or exposure, should have routine upfront coverage for this organism.^3^

Second, empiric combination therapy with a macrolide should be considered in patients admitted with severe CAP, both because severe infection may indicate a higher risk for *Legionella* and given evidence suggests a possible immunomodulatory effect of macrolides in severe CAP.^10^ In comparison to other atypical pathogens, patients with pneumonia due to *Legionella* often present with severe presentations and empiric bacterial coverage in this high-risk population is reasonable until additional diagnostic data is available to guide further treatment decisions.^10,49^

Third, findings from two RCTs suggest that in a subset of general medicine ward patients with more severe CAP presentations not requiring ICU care (patients with PSI category IV pneumonia), the addition of clarithromycin to β–lactam therapy may provide a favorable impact on clinical response rates (without a mortality benefit) by attenuating the inflammatory burden.^18,23^ The benefit of immunomodulation in severe CAP presentations is consistent with other literature noting a mortality benefit of hydrocortisone in severe CAP.^50^ Despite these findings, judicious use of macrolides should be considered in this population when considering their role in pneumonia (immunomodulatory versus antibacterial), and when weighed against the potential risks of use, particularly in light of increasing macrolide resistance and associated harms.

Fourth, patients with immunocompromising conditions were excluded in 2/3 of the referenced RCTs above.^18,23,24^ In the study conducted by Postma and colleagues, 17% (n = 381/2,283) of the included population had a coexisting condition listed as solid or hematologic cancer; unfortunately, outcomes were not compared to immunocompetent counterparts.^24^ The prevalence of atypical infections in immunocompromised patients and benefit of immunomodulation compared to immunocompetent individuals are important factors that should be considered. In 2015, Pasquale and colleagues performed an international, multicenter study in 222 hospitals across 54 countries to assess the etiology of CAP among immunocompromised adults hospitalized with CAP, defined as the presence of hematological cancer, chemotherapy in the last 3 months, neutropenia, biological drug use, lung transplantation, chronic steroid use, solid tumor with either neutropenia or chemotherapy, acquired immunodeficiency syndrome (AIDS), aplastic anemia, and asplenia.^51^ Among patients that underwent microbiological testing, 596 were immunocompromised and 2,626 were immunocompetent. There was no difference in the prevalence of atypical pathogens (1.7% vs 1.9%, *P* = 0.78) between study arms. Sivagnanam and colleagues also conducted a retrospective study to understand the epidemiology of Legionnaires’ disease in both solid organ transplant and hematopoietic cell transplant recipients and found that 0.8% of transplant recipients who underwent *Legionella*-specific testing by Legionella culture and/or urinary antigen testing from 1999 to 2013 were positive (n = 32), further suggesting that the low prevalence of this pathogen in pneumonia is similar to the general population.^52^ Among studies that have assessed *Legionella* pneumonia in immunocompromised patients, *L. pneumophila* remains the most implicated species of *Legionella* infections in adults, even among immunocompromised patients; overall, non-pneumophila *Legionella* infections are rare.^52–54^ Additionally, there are insufficient data to address the benefit of immunomodulation among immunocompromised patients. Guidance on management strategies for immunocompromised patients suggests targeting core respiratory pathogens for adults hospitalized with non-severe CAP and initial standard empiric antibacterial recommendations are consistent with those for immunocompetent patients,^55^ which currently includes combination therapy with a macrolide. In the absence of clinical data, it is reasonable to continue this approach. However, given the low prevalence of *Legionella* infections demonstrated among immunocompromised adults, it would also be reasonable to consider the suggested treatment approach for immunocompetent adults with non-severe CAP outlined below.

### Suggested approach for empiric antibacterial coverage in non-severe hospitalized patients with CAP (Table 4)

For the majority of patients hospitalized with non-severe CAP, we suggest that standard empiric treatment should include β–lactam monotherapy (Table 4). As empiric β–lactam-macrolide combination therapy may only benefit non-severe CAP patients with features of sepsis or those with risk factors for *Legionella*, we would reserve upfront macrolide therapy for these individuals outside the ICU.

**Table 4.** Initial treatment strategies for inpatients with non-severe community-acquired pneumonia (CAP)

Standard regimen	Preferred: β–lactam monotherapy
	Alternative: Respiratory fluoroquinolone monotherapy
SOFA score ≥2, risk factors for *Legionella*^a^, +/− severe immune compromise^b^	Preferred: β–lactam + macrolide
	Alternative: β–lactam + doxycycline (long QTc)

a Risk factors for *Legionella*: travel to Europe in summer months, environmental water exposure (pools, hot tubs, birthing pools, cooling towers, tropical storms), association with a known *Legionella* outbreak.

b Severe immune compromise: active neutropenia, solid organ transplant or hematopoietic stem cell transplant recipients, patients on prednisone ≥20 mg daily or equivalent for ≥2 weeks, or acquired immunodeficiency syndrome (AIDS).

## Summary

In conclusion, routine empiric atypical antibacterial coverage may not be necessary for all non-severe patients hospitalized with CAP. Among existing data including well-conducted RCTs, combination therapy with a macrolide has not been associated with a mortality benefit but may decrease time to clinical stability in those with non-severe CAP and PSI category IV pneumonia. These findings taken together with the increased development of macrolide resistance, risks of cardiac events and other harms, and low incidence of atypical pathogens in pneumonia suggest we should reconsider the routine empiric addition of macrolides to standard inpatient treatment regimens for CAP.
